# Supplementary material for: Correlation between Peptacetobacter hiranonis, the baiCD Gene, and Secondary Bile Acids in Dogs
Source: Animals (Basel). 2024 Jan 9;14(2):216. doi: 10.3390/ani14020216 (PMC10812727; doi:10.3390/ani14020216)
Supplement: Supplementary file 1 [file animals-14-00216-s001.zip › animals-2726041-supplementary.pdf]

Supplementary Table S1

Table S1. The amplicon length, melting peak temperature, efficiency of the qPCR assay and the R^2 of the calibration curve for the *baiCD* qPCR assay.

Target	Amplicon Length (bp)	Melting Temperature (◦C)	Efficiency* (%)	R^2 of Calibration Curve
baiCD gene	721	80.0	91.6	0.994

*The theoretical efficiency is calculated by $E = 10^{-1/slope}$

Supplementary Table S2

Table S2. Specificity of the *baiCD* primers against other bacterial species

**Bacterial Species**	**Amplification**
Clostridium difficile	Not detected
Clostridium scindens	Not detected
Escherichia coli	Not detected
Akkermansia muciniphila	Not detected
Faecalibacterium duncaniae	Not detected

Supplementary Table S3

Table S3. Formula for calculating the relative concentration (%) of fecal unconjugated bile acids in fecal dry matter.

**fUBA**	**Formula**
Primary fUBA (%)	Primary fUBA (%) = Total Primary fUBA (CA and CDCA) / Total fUBA (CA, CDCA, UDCA, LCA and DCA)) * 100
Secondary fUBA (%)	Secondary fUBA (%) = Total Secondary fUBA (LCA, DCA and UDCA) / Total fUBA (CA, CDCA, UDCA, LCA and DCA)) * 100
